# Morphological, morphometrical, and molecular characterization of *Metarhabditis amsactae* (Ali, Pervez, Andrabi, Sharma and Verma, 2011) Sudhaus, 2011 (Rhabditida, Rhabditidae) from India and proposal of *Metarhabditis longicaudata* as a junior synonym of *M. amsactae*

**DOI:** 10.21307/jofnem-2020-116

**Published:** 2020-12-14

**Authors:** Aashaq Hussain Bhat, Shreyansh Srivastava, Aasha Rana, Ashok Kumar Chaubey, Ricardo A. R. Machado, Joaquín Abolafia

**Affiliations:** 1 Nematology Laboratory, Department of Zoology, Chaudhary Charan Singh University, Meerut, India; 2 Experimental Biology Research Group, Institute of Biology, University of Neuchâtel, 2000, Neuchâtel, Switzerland; 3 Departamento de Biología Animal, Biología Vegetal y Ecología, Universidad de Jaén, Jaén, Spain; 4 Government Degree College, Billawar, 184204, Kathua, Jammu and Kashmir, India

**Keywords:** 18S rDNA, 28S rDNA, ITS rDNA, *Metarhabditis amsactae*, *Metarhabditis longicaudata* n. syn., Molecular analysis, Morphology, New synonym, Taxonomy

## Abstract

A new population of *Metarhabditis amsactae* from India is morphologically, morphometrically, and molecularly characterized. This material is characterized by having 0.65 to 1.14 mm length, lips rounded, and grouped in pairs, stoma with metastegostoma bearing setose denticles, pharynx with metacorpus slightly swollen and fusiform, nerve ring, and excretory pore located at isthmus level, female reproductive system didelphic-amphidelphic with vulva equatorial, female tail conical-elongate with acute tip, male tail conical with large and robust posterior filiform part, spicules free with hooked manubrium slightly bent ventrad, gubernaculum with narrow corpus, bursa open leptoderan with eight genital papillae and phasmids posterior to the GP8. Molecular studies based on 18S and 28S rDNA genes are provided for the first time for the species. In addition, integrated morphological, morphometrical, and molecular characters are compared with other previous records of the species. According to our analysis, *Metarhabditis longicaudata* and other material described as different species are proposed as new junior synonyms of *M. amsactae*.

The genus *Metarhabditis* Tahseen, Hussain, Tomar, Shah and Jairajpuri, 2004 was proposed by Tahseen et al. (2004) under the family Rhabditidae Örley, 1880 with the type and only species *Metarhabditis andrassyana* Tahseen, Hussain, Tomar, Shah and Jairajpuri, 2004. This genus is characterized by having metastegostom with knobbed setose denticles and bursa bearing eight genital papillae. The genus was later revised by Sudhaus (2011) who transferred five species from the genera *Rhabditis* Dujardin, 1845 namely *Rhabditis adenobia* Poinar, 1971, *R. blumi* Sudhaus, 1974, *R. costai* Martins, 1985, *R. freitasi* Martins, 1985, and *R. rainai* Carta and Osbrink, 2005 and one species from *Oscheius* Andrássy, 1976 namely *Oscheius amsactae* Ali, Pervez, Andrabi, Sharma and Verma, 2011 into *Metarhabditis*. Recently, Abolafia and Peña-Santiago (2019a) described a new species, *M. giennensis* from Spain and provided a key for species identification. More recently, Tabassum et al. (2019) described other new species, *M. longicaudata* from Pakistan and its identity is discussed later in this paper.

One of the species recently transferred to the genus *Metarhabditis* is *M. amsactae* (Ali, Pervez, Andrabi, Sharma and Verma, 2011) Sudhaus, 2011. It is distinguished from all the other species of the genus by having large and robust posterior filiform part of the male tail (see keys to species identification provided by Abolafia and Peña-Santiago, 2019a). It was first described as *Oscheius amsactae* by Ali et al. (2011), who recovered some nematode specimens from a larva of the red-hairy caterpillar, *Amsacta moori* Butler (Lepidoptera: Arctiidae), collected in Kanpur, Uttar Pradesh, India. Since then, *M. amsactae* nematodes have been isolated from soil samples in different regions of India and Pakistan, many of them were previously identified as other species, however, such as *Oscheius ciceri* Shaheen, Ali and Asif, 2011, *Oscheius hussainii* Shaheen, Ali and Asif, 2011, *Oscheius gingeri* Pervez, Eapen, Devasahayan, and Jacob, 2012, and *Oscheius amsactae* Ali, Pervez, Andrabi, Sharma and Verma, 2011 and *Metarhabditis longicaudata* Tabassum, Salma and Nasir, 2019. Most of these studies, however, have characterized the species morphologically and morphometrically. Regarding molecular analysis, several Internal Transcribed Spacer (ITS) rDNA sequences, obtained from *M. amsactae* isolated in India, Philippines, and Pakistan have been deposited in the GenBank, but none of the nematode specimens used to obtain the sequences were morphologically characterized and vice versa. Hence, scanning electron microscopy images and reference molecular data for this species are still required. In this study, we therefore conducted the scanning electron microscopy (SEM) studies, and sequenced the ITS, and small-subunit (SSU) and large-subunit (LSU) rDNA of two *M. amsactae* isolated from Uttar Pradesh, India.

## Materials and methods

### Nematode sampling

A survey to obtain the nematodes was conducted in soils of the district Shamli (29.6189° N, 77.4329° E; 280  m altitude), Uttar Pradesh, India. This location has a semiarid and moderate-to-tropical monsoon (humid subtropical) predominant climate. The type of soil is sandy loam and loamy and the pH of soil samples ranged from 6.5 to 8.4. A total of eighty-nine soil samples were taken from meadows, pastures, agricultural fields, open fields, and orchards.

Each soil sample consisted of 1 kg of soil that was a mixture of five soil subsamples collected at 15 to 20  cm depth in five locations within each field (one sample from each corner of the field, and one from the center of the field). The soil was first made fine to remove any debris (i.e., rocks, pieces of wood or bark, leaves, etc.) and then moistened with distilled water using a spray bottle to facilitate the movement of nematodes. To recover insect-associated nematodes from these soil samples, the ‘*Galleria mellonella* baiting’ method and the White (1927) trap method modified (Bedding and Akhurst, 1975) were used. Seven 4th instar *Galleria mellonella larvae* were buried in 250 ml of autoclaved plastic containers filled with the collected soil up to the brim. The plastic containers were then covered with tissue paper and muslin cloth. The containers were inverted upside-down and stored in the dark in an incubator at 27 ± 2°C for 7 days. The plastic containers were checked daily to recover dead insect larvae. Insect cadavers were rinsed with double-distilled water (ddH_2_O) to remove soil particles and disinfected with 0.1% sodium hypochlorite before being placed on the modified White traps to obtain emerging infective juveniles. The White traps were incubated at 27 ± 2°C in an incubator and checked daily for the emergence of third-stage juveniles from the cadavers. Emerged third-stage juveniles migrate after 5 to 7 days to water surrounding the Petri dish and nematodes were collected regularly until nematode emergence ceased after 10 to 20 days (Bhat et al., 2018, 2019).

Emerged IJs were sterilized with 0.1% sodium hypochlorite and washed with ddH_2_O, and finally stored in tissue culture flasks at 15°C. Third-stage juveniles were used within seven days after emergence (Aasha et al., 2019; Bhat et al., 2020a).

### Nematode morphology and morphometry

Nematode third-stage juveniles were surface-sterilized with 1% NaOCl. Then, greater wax moth (*Galleria mellonella*) larvae were injected with 100 juvenile nematodes in sterile Petri plates using a 1 ml of insulin syringe. The male, female, and juvenile (third-stage) nematode generation were recovered from White traps as described above. All nematode generations were heat-killed in Ringer’s solution and fixed in triethanolamine formalin (Courtney et al., 1955). Nematodes were infiltrated in glycerol by the Seinhorst method (Seinhorst, 1959) and processed further as described by Bhat et al. (2017). Briefly, the nematodes were kept in pure glycerol. Three females, specimens of five males, and 10 infective juvenile nematodes were mounted separately in a drop of glycerol on a clean glass slide. Paraffin wax was used to seal and to prevent the flattening of nematode specimens (Bhat et al. 2020b; Kajol et al., 2020). The morphology and morphometric analysis of the specimens was conducted using light compound microscope (Magnus MLX) and phase-contrast microscope (Nikon Eclipse 50i). Twenty specimens of adults (male and female) and 20 of juveniles were analyzed. Morphometric analyses were carried out with the aid of in-built software of the phase-contrast microscope (Nikon DS-L1). The best-preserved specimens were also photographed using a Nikon Eclipse 80i (Nikon, Tokyo, Japan) light microscope provided with differential interference contrast optics (DIC) and a Nikon Digital Sight DS-U1 camera. Micrographs were edited using Adobe® Photoshop® CS. Nematode species were identified based on morphological and morphometric characters using the key provided by Abolafia and Peña-Santiago (2019a). Demanian indices (de Man, 1881) and other ratios were calculated. The terminology used for the morphology of the stoma and spicules/gubernaculum follows the proposals by De Ley et al. (1995) and Abolafia and Peña-Santiago (2017), respectively.

### Scanning electron microscopy (SEM)

For the SEM, male and female specimens preserved in glycerin were selected for observation and processed according to the Abolafia’s (2015) protocol. Thus, they were hydrated in distilled water, dehydrated in a graded mixture of ethanol-acetone series, critical point-dried with liquid carbon dioxide, and coated with gold. The mounts were examined with a Zeiss Merlin microscope (5 kV).

### Nematode molecular characterization

Genomic DNA was isolated from approximately five hundred infective juvenile nematodes by using the Qiagen Blood and Tissue Analysis Kit following the manufacturer’s protocol. A fragment of the rDNA gene containing the ITS regions (ITS1, 5.8S, ITS2) was amplified using primers 18S: 5′-TTGATTACGTCCCTGCCCTTT-3′ (forward), and 26S: 5′-TTTCACTCGCCGTTACTAAGG-3′ (reverse) (Vrain et al., 1992). The fragment containing the D2/D3 regions of the 28S rDNA gene was amplified using primers D2F: 5′-CCTTAGTAACGGCGAGTGAAA-3′ (forward) and 536: 5′-CAGCTATCCTGAGGAAAC-3′ (reverse) (Nadler et al., 2006). The 18S rDNA was amplified using primers NEM18SF: 5′-CGCGAATRGCTCATTACAACAGC-3′ (forward) and NEM18SR: 5′-GGGCGGTATCTGATCGCC-3′ (reverse) (Floyd et al., 2005). The Polymerase Chain Reaction (PCR) protocol for ITS, 18S, and D2/D3 rDNA gene amplification followed was described by Bharti et al. (2020) and Suman et al. (2020). Briefly, PCR master mix consisted of ddH_2_O 16.8 μl, 10x PCR buffer 2.5 μl, dNTP mix (10 mM each) 0.5 μl, 1 μl of each forward and reverse primers, dream taq green DNA polymerase 0.2 μl, and 3 μl of DNA extract. The PCR profiles used were 1 cycle of 94°C for 3 min followed by 40 cycles of 94°C for 30 sec, 52°C for 30 sec for LSU (28S) rDNA or 55°C for 30 sec for ITS rDNA or 54°C for 30 sec for SSU (18S) rDNA, 72°C for 60 sec, and a final extension at 72°C for 10 min. PCR was followed by electrophoresis (45 min, 100 volts) of 5 μl of PCR product in a 1% TAE (Tris–acetic acid–EDTA) buffered agarose gel stained with ethidium bromide (Bhat et al., 2020c; Rana et al., 2020a). The amplified products were purified and Sanger sequenced in both directions by Bioserve Technologies Ltd. (Hyderabad, India). The obtained sequences were manually curated, trimmed, and submitted to the Center for Biotechnology Information (NCBI) under accession numbers, MT873043, MT872508, and MT872503 for ITS, 28S (D2/D3) and 18S rDNA regions, respectively for the isolate CJ6, and MT873044, MT872509, and MT872504 for the same respective genes for the isolate CJ13.

### Sequence alignment and phylogenetic analyses

The sequences were edited and compared with those already present in GenBank using the Basic Local Alignment Search Tool (BLASTN) of the National Center for Biotechnology Information (NCBI) (Altschul et al., 1990). The newly obtained ribosomal LSU (D2/D3 rDNA), SSU (18S rDNA), and ITS (ITS1, 5.8S, ITS2) rDNA sequences were manually edited using BioEdit 7.2.6 (Hall, 1999) and aligned with other relevant segments of same rDNA gene sequences available in GenBank using Clustal W alignment in the program MEGA7 (Kumar et al., 2016). Poorly aligned regions were removed from the alignments using MEGA7. The base substitution model was evaluated using jModeltest2.1.10 (Darriba et al., 2012). Phylogenetic trees were elaborated using the Bayesian inference method as implemented in the program MrBayes 3.2.7 (Ronquist et al., 2012). For analysis in jModeltest, the HKY + I + G model was selected for the ITS tree, the GTR + I + G model was selected for the 18S tree, and the GTR + G was selected for the 28S tree. The selected models were initiated with a random starting tree and ran with the Markov chain Monte Carlo (MCMC) for 1 × 10^6^ generations. The Bayesian tree was ultimately visualized using the FigTree program 1.4.4 (Rambaut, 2018). *Heterorhabditis downesi* (KU573061) was used as the outgroup and to root the trees for ITS1-5.8S-ITS2 rDNA tree, *Myolaimus byersi* (KU180676) for LSU rDNA tree, and *M. byersi* (KU180665) for SSU rDNA tree.

The details of all the nematode species used in the molecular and phylogenetic study, including their updated nomenclature, accession numbers of rDNA genes, isolation source, and origin of the sequences are given in Table 4.

## Results

### Insect-associated nematode isolation

Nematodes of four genera: *Metarhabditis*, *Steinernema, Heterorhabditis,* and *Oscheius* were recovered from the eighty-nine soil samples collected in this study. Two soil samples, taken around the rhizosphere of sugarcane (*Saccharum officinarum* L.) and groundnut (*Arachis hypogaea* L.), contained *Metarhabditis* nematodes. Five samples were found positive for the presence of *Steinernema abbasi*, two for the presence of *Heterorhabditis indica,* and two for the presence of *Oscheius* sp. The rest of the samples were found negative for the presence of insect-associated nematodes. In this study, we characterized *Metarhabditis* nematodes. *Steinernema, Heterorhabditis,* and *Oscheius* nematodes are characterized in other studies (Bhat et al., 2020c, Rana et al., 2020b).

### Systematics

#### Metarhabditis amsactae

**Figure 1:**
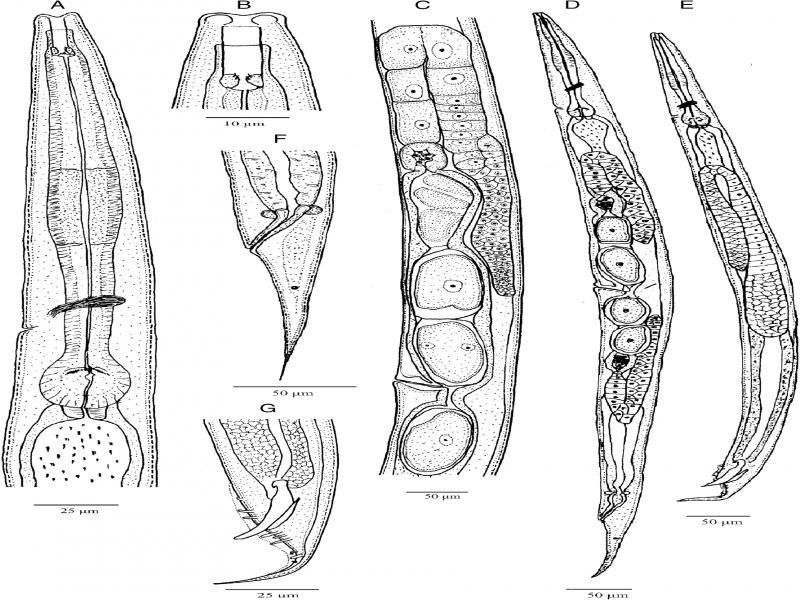
*Metarhabditis amsactae* (Ali, Pervez, Andrabi, Sharma and Verma, 2011) Sudhaus, 2011 (line drawing). A: Anterior region; B: Cephalic region; C: Anterior branch of the female reproductive system; D: Entire female; E: Entire male; F: Female posterior region; G: Male posterior region.

**Figure 2:**
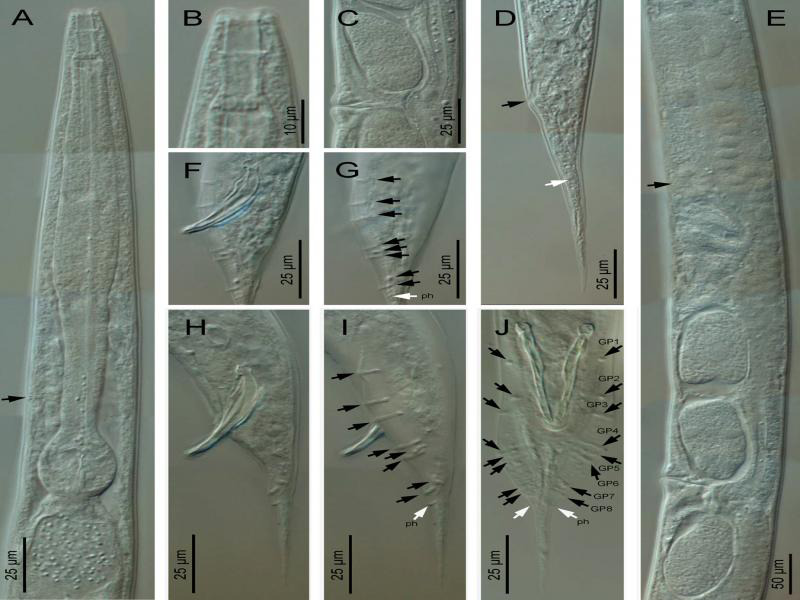
*Metarhabditis amsactae* (Ali, Pervez, Andrabi, Sharma and Verma, 2011) Sudhaus, 2011 (light microscopy). A: Anterior region (arrow pointing to the excretory pore); B: Cephalic region; C: Vagina region; D: Female posterior region (black arrow pointing to the anus, white arrow pointing to the phasmid); E: Anterior branch of the female reproductive system (black arrow pointing to the spermatheca); F-J: Male posterior region in lateral (F-I) and ventral (J) views, at spicules (F, H) and bursa (G, I) level (black arrows pointing to the genital papillae, white arrows pointing to the phasmids).

**Figure 3:**
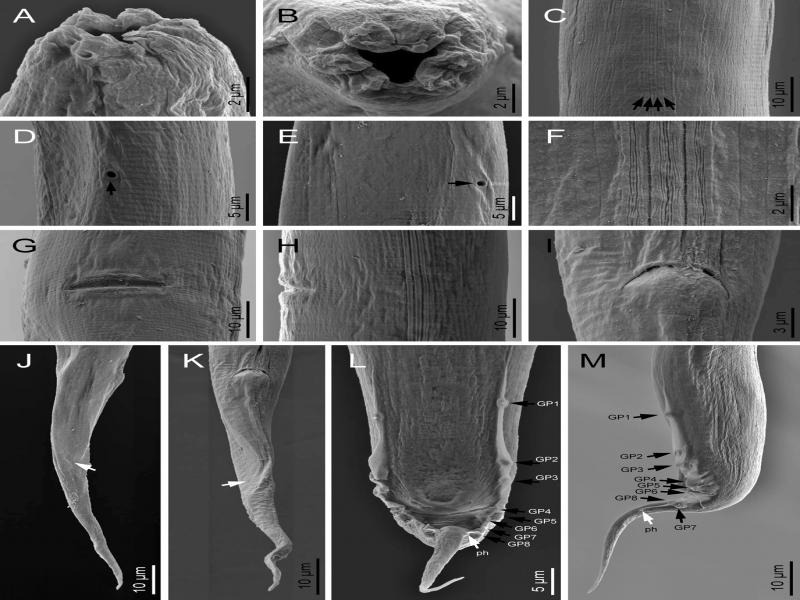
*Metarhabditis amsactae* (Ali, Pervez, Andrabi, Sharma and Verma, 2011) Sudhaus, 2011 (scanning electron microscopy). A, B: Cephalic and lip region in sublateral and frontal views, respectively; C, F: Lateral field (arrows pointing to the longitudinal incisures); D, E: Excretory pore (arrow) at ventral and lateral views, respectively; G, H: Vulva in ventral and lateral views, respectively; I: Female anus; J, K: Female posterior region in right lateral and ventral views, respectively (white arrows pointing to the phasmid); L, M; Male posterior region in ventral and lateral views (black arrows pointing to the genital papillae, white arrows pointing to the phasmids).

(Ali, Pervez, Andrabi, Sharma and Verma, 2011) Sudhaus, 2011.

(Figs. 1-3 and Table 1)

**Table 1.** Morphometrics of *Metarhabditis amsactae* (Ali, Pervez, Andrabi, Sharma and Verma, 2011) Sudhaus, 2011 from India.

Characters	Female	Male	L3
n	20	20	20
Body length (*L*)	939 ± 119 (718-1135)	800 ± 91 (653-999)	383 ± 55 (305-475)
*a* (*L*/MBW)	16.4 ± 2.7 (10.9-21.1)	16.7 ± 2.0 (12.7-20.7)	18.9 ± 2.0 (15.7-22.9)
*b* (*L*/NL)	5.4 ± 0.6 (4.5-6.6)	4.9 ± 0.5 (3.8-5.9)	3.4 ± 0.5 (2.7-4.3)
*c* (*L*/T)	10.9 ± 1.7 (8.7-14.0)	13.7 ± 2.7 (9.8-20.9)	7.5 ± 1.2 (6.0-10.2)
*c′* (*T*/ABW)	4.1 ± 0.6 (2.9-5.2)	2.9 ± 0.5 (1.5-3.8)	4.8 ± 1.0 (3.0-6.0)
*V* (VA/*L* × 100)	51.2 ± 2.1 (46-56)	–	–
Lip region width	7.9 ± 1.4 (5-11)	7.1 ± 1.1 (6-10)	3.3 ± 0.5 (2-5)
Stoma length	18.4 ± 2.3 (14-22)	17.1 ± 2.3 (13-21)	12.8 ± 1.1 (11-15)
Stomatal tube width	5.0 ± 0.4 (2.5-3.5)	3.5 ± 0.7 (2.5-5.5)	?
Pharyngeal corpus length	74 ± 5.6 (68-98)	68 ± 4.5 (58-76)	35 ± 2.9 (30-42)
Metacorpus length	31 ± 3.9 (24-36)	30 ± 2.6 (24-33)	22 ± 2.4 (19-27)
Isthmus length	40 ± 5.5 (40-48)	39 ± 2.5 (35-41)	27 ± 2.6 (23-31)
Bulb length	29 ± 2.3 (26-34)	29 ± 2.6 (24-35)	16.8 ± 2.8 (12-24)
Pharynx length	175 ± 13.3 (156-195)	166 ± 7.3 (141-175)	101 ± 6.5 (88-112)
Nerve ring – anterior end	123 ± 15.0 (98-153)	112 ± 9.1 (92-125)	69 ± 10.9 (53-92)
Excretory pore–anterior end (EP)	137 ± 18.2 (110-166)	130 ± 9.1 (113-144)	78 ± 11.7 (56-103)
Deirid–anterior end	133 ± 16.0 (110-167)	125 ± 11.0 (107-147)	?
Neck length (stoma + pharynx, NL)	173 ± 15.0 (148-195)	163 ± 8.3 (144-176)	114 ± 6.1 (101-124)
Body width at neck base	43 ± 5.2 (32-50)	38 ± 4.2 (32-50)	18.7 ± 2.9 (14-25)
Mid-body width (MBW)	58 ± 10.4 (43-81)	48 ± 7.2 (40-66)	20.5 ± 3.3 (16-29)
Anterior genital branch or Testis	260 ± 38.2 (192-372)	198 ± 15 (188-222)	–
Posterior genital branch	278 ± 27.5 (229-321)	–	–
Vagina length	24.5 ± 4.1 (17-30)	–	–
Vulva–anterior end (VA)	480 ± 64 (380-579)	–	–
Rectum length	31 ± 6.4 (22-42)	–	15.0 ± 4.0 (9-23)
Anal body width (ABW)	22 ± 2.7 (16-28)	20.9 ± 2.5 (17-27)	11.2 ± 2.2 (9-17)
Tail length (T)	87 ± 10.5 (68-101)	63 ± 8.2 (49-62)	52 ± 5.9 (48-58)
Spicules length	–	41 ± 7.5 (34-49)	–
Gubernaculum length	–	19.6 ± 3.5 (16-28)	–

**Notes:** = Character absent. ? = Measurement unknown. Measurements in μm (except *n*, ratio, and percentage) and in the form: mean±standard deviation (range).

### Description

*Adult*: Body 0.72 to 1.14  mm long in females and 0.65 to 1.00  mm long in males, mostly straight rarely arcuate upon gentle heat killing with tapering to the anterior and posterior ends, more tapering toward the posterior end. The cuticle striated with scarcely prominent annuli 1.0 to 1.5  µm wide varying with body regions. Lateral fields were indistinct under light microscopy; however, four longitudinal lines are visible under scanning electron microscopy. The Lip region was almost continuous from contiguous body. Lips rounded and swollen, organized in doublets forming three pairs (one dorsal and two subventral) around the triradial oral orifice. Amphids small, oval, positioned at the base of lateral lips. Stoma rhabditoid type, 1.5 to 3.4 times the lip region width in length, with stomatal tube (gymno-promesostegostom) well developed. Cheilostom short with poorly refringent rhabdia; gymnostom tubular with cuticularized rhabdia, shorter than promesostegostom, this later surrounded by a thin pharyngeal collar; metastegostom isomorphic and isotopic having glottoid apparatus with three valves bearing two setose denticles per valve; telostegostom with minute rounded rhabdia. Pharynx rhabditoid, differentiated into cylinder-shaped pharyngeal corpus, 0.9 to 2.0 times the isthmus length, metacorpus slightly swollen, fusiform, isthmus relatively thick, weakly narrowing until its junction with the basal bulb, this more or less rounded, occasionally pyriform, with a weak to moderately developed valvular apparatus and faintly double-chambered haustrulum. Nerve ring surrounding the pharynx at the level of isthmus, 83 to 89% of neck length. Secretory–excretory pore at 79 to 86% of neck length, variable in position ranging from middle of isthmus to closely anterior to basal bulb. Deirids and hemizonid poorly visible, posterior to excretory pore, at 84 to 96% of neck length, at level of isthmus. Cardia small, conoid, surrounded by intestinal tissue. Intestinal lumen wider and dilated posterior to the basal bulb.

Female: Reproductive system didelphic-amphidelphic, the anterior and the posterior branches in sinistral and dextral position to intestine, respectively. Ovaries moderately developed, dorsally reflexed but with distal end not reaching to vulval level, anterior ovary slightly larger. Usually one or two small rounded pseudocoelomocytes observed in close proximity to the proximal end of ovaries. Oviducts proximally enlarged, connected to ovoid spermatheca frequently filled with sperm. Uteri well developed, differentiated into long glandular and muscular regions, filled with sperm and one to ten intrauterine eggs, 40−49 × 22−23 µm, in different stages of embryonation. Vagina thick-walled, often cuticularized, at right angle to the longitudinal body axis, with length equal to about one-third of the vulval body diameter. Vulva a wide transverse slit, with protruding lips, unremarkable or weak epiptygma but distinct cuticular flap. Rectum short, 1.0 to 1.8 times anal body diameter, allied with rectal glands at its junction with prerectum. Prerectum distinguishable from intestine in lacking prominent cell nuclei. Tail elongate conoid, gradually tapering to a fine terminus. Phasmids short tubular, located posterior to anus, about 38 to 42% of tail length.

Male: Similar to female in general morphology except for smaller size, posterior body curvature prominent and cuticular striations relatively fine. Reproductive system monorchic, with single testis reflexed ventrad anteriorly on the right side of the intestine. Vas deferens a broad tube, packed with sperm without delineation of seminal vesicles. Ejaculatory glands not observed. Spicules paired and symmetrical, ventrally arcuate, free with slightly bent ventrad manubrium, ventrally hooked, calamus short conoid and slightly ventrally curved lamina with ventrally bent finely rounded tip in lateral view. Gubernaculum well-developed, slightly ventrad curved with long manubrium and narrow corpus, 50 to 60% of spicule length. Three small gland-like cells are distinguishable around the anterior end of the cloaca. Tail conoid with posterior two-thirds abruptly tapering and reduced. Bursa anteriorly open, narrow, leptoderan, not enclosing large tail spike, having smooth margins and eight pairs (1 + 1 + 1/3 + 2 + ph) of genital papillae, with GP1 and GP2 spaced, precloacal, GP3 slightly posterior to cloaca in most specimens, pairs GP4 to GP6 located at conoid part of tail and GP7 to GP8 located at posterior part of the bursa, dorsally directed. Phasmid small, tubular, located posterior to the GP8, at 45 to 50% of tail length.

Juveniles: Third-stage juveniles ensheathed in a cuticle of second stage juveniles. Sheath free anteriorly in third-stage juveniles, firmly bound to the posterior region of the body. Body lean, from anus to tail terminus. Cuticle with transverse striae. Lip region smooth; stoma opening closed. Stoma tubular. Pharynx with pharyngeal corpus and isthmus both long and narrow, and basal bulb spheroid, valvate. Nerve ring and excretory pore located at isthmus level. Tail conoid with pointed terminus.

### Molecular characterization

From the two populations of *Metarhabditis amsactae* molecularly analyzed in the present study from India, two sequences of 18S rDNA (865 and 869 bp), two of D2/D3 fragment of 28S rDNA (887 and 907 bp) and two of ITS1-5.8S-ITS2 rDNA (885 and 883 bp) have been obtained. Sequences of 18S and 28S rDNA are obtained for the first time for this species. A common aligned fragments resulted in 865 bp for the 18S rDNA, 879 bp for the 28S rDNA and 883 bp for the ITS rDNA, any of them show changes (substitutions, deletions or insertions) in their respective sequences.

Comparing with other sequences (unpublished) of the species available from GenBank, the 18S rDNA fragment, from a common aligned fragments with 794 bp, the present populations from India shows one (0.1%) change from the other sequence available from India (NM453373), 1 (0.1%) and 16 (2.0%) changes from the sequences submitted from Philippines (MT012150 and MT043860), respectively. For the 28S rDNA fragment, there are no other available sequences to compare. The ITS rDNA sequences, from a common aligned fragments with 645 bp, show 3 (0.5%) or 6 (0.9%) changes from other sequences submitted from India (KP834432/KP834433/KY083045 and MH392568), respectively; 1 (0.2%) or 2 (0.3%) changes with respect to two sequences from Philippines (MT422254 and MT576957), while other two sequences (MT452472 and MT576957) deposited from Philippines show too much changes (51 and 64, respectively); the sequence submitted from Pakistan (MK973071) show 24 (3.7%) changes, the most of them consistent in two long contiguous deletions in the Pakistani sequence (10 and 12 gaps, respectively, after aligning sequences), which must be considered as *M. amsactae*.

### Voucher material

Twenty females and twenty males of each isolate were deposited at the museum of the Department of Zoology, Chaudhary Charan Singh University Meerut, India. Ten females and ten males were deposited at the nematode collection of the Department of Animal Biology, Plant Biology and Ecology of the University of Jaén, Spain.

### Diagnosis (based on the species and its synonyms)

*Metarhabditis amsactae*, including its synonyms, are characterized by having a body length of 0.72 to 2.07 mm in females and 0.65 to 1.50 mm in males, cuticle with very fine transverse striations; lips rounded and swollen grouped in pairs, stoma with metastegostoma bearing setose denticles, esophagus with metacorpus slightly swollen and fusiform, nerve ring and excretory pore located at isthmus level, female reproductive system didelphic-amphidelphic with vulva equatorial (*V* = 42-60), female tail conical-elongate with acute tip (65-148 µm long, *c* = 8.7-18.0, *c′* = 2.5-8.0), female phasmids located about the middle length of the tail, male tail conical (32-76 µm long, *c* = 9.8-37.0, *c′* = 1.0-3.8) with large and robust posterior filiform part, spicules free (24-60 µm long) with rounded manubrium slightly bent ventrad and hooked ventrally, gubernaculum 9-34 µm long, bursa open leptoderan with eight genital papillae (1 + 1/1/3 + 2 + ph) and phasmids posterior to the GP8.

### Remarks

The material examined in this study agrees well with the type material described by Ali et al. (2011) and the redescription of Asif et al. (2013) as *M. amsactae*. Morphologically, the present material does not show important morphological differences with previous described populations. With respect to other populations described from different geographical regions of India (Shaheen et al., 2011; Pervez et al., 2012 as *Oscheius ciceri* and *O. hussaini*; Asif et al., 2013 as *Oscheius gingeri*), the material examined now shows close similitude to each other with only variations in body length, pharyngeal corpus, and isthmus length in adult generations (see Tables 2 and 3). The variation in morphometry in the present Indian population compared with the other populations can be attributed to differences in their geographical origin.

**Table 2.** Compendium of females of *Metarhabditis amsactae* (Ali, Pervez, Andrabi, Sharma and Verma, 2011) Sudhaus, 2011 populations and its synonyms.

Species	*M. amsactae*	*M. amsactae*	*M. amsactae* as *O. ciceri*	*M. amsactae* as *O. hussainii*	*M. amsactae* as *O. gingeri*	*M. amsactae*	*M. amsactae* as *M. longicaudata*	*M. amsactae* as *M. rainai*	*Metarhabditis* sp. as *M. amsactae*
Reference	Present study	Ali et al. (2011)	Shaheen et al. (2011)	Shaheen et al. (2011)	Pervez et al. (2012)	Asif et al. (2013)	Tabassum et al. (2019)	Tabassum et al. (2019)	Tabassum et al. (2019)
Country	India	India	India	India	India	India	Pakistan	Pakistan	Pakistan
Habitat	Rhizosphere of sugarcane and groundnut	Rhizosphere of mungbean	Rhizosphere of chickpea	Rhizosphere of pigeonpea	Rhizosphere of ginger	Decaying matter	Rhizosphere of mango tree	Decomposed guava fruit	Rhizosphere of chicko
*L*	718-1,135	658-786	964-1,018	902-989	1,418-1,813	786-902	1,366-1,684	1,769-2,078	1,546-1,694
*a*	10.9-21.1	19.7-22.9	20.1-22.5	24.4-25.5	18.5-21.2	19.2-23.5	14.0-18.0	11.7-20.0	15.0-18.0
*b*	4.5-6.6	4.1-4.8	5.7-5.9	3.8-4.3	5.1-5.3	4.1-5.0	5.5-7.8	7.0-8.0	6.0-8.0
*c*	8.7-14.0	8.9-12.1	10.3-12.9	10.2-12.7	12.1-13.2	9.6-11.2	12.0-16.0	12.0-18.0	13.0-19.0
*c′*	2.9-5.2	4.1-4.6	3.6-4.3	3.3-4.8	4.6	3.8-4.5	2.5.-4.4	4.0-8.0	3.0-4.0
*V*	46-56	50-58	43^a^	42^a^	51-60	51-55	48-56	49-58	50-54
Lip region width	5-11	7-8	8-11	6-8	8-12	9-10	11-15	12^a^	12^a^
Stoma length	14-22	16-18	18-19	22-23	19-21	20-25	22-28	26-30	22-26
Corpus length	68-98	96-115	92-100	133	95-170	50^a^	62-106	57^a^	42^a^
Isthmus length	40-48	32-44	30-40	44-56	28-57	35-45	30^a^	27^a^	33^a^
Bulb length	26-34	24-35	26-31	33-45	44^a^	25-35	19^a^	16^a^	17^a^
Nerve ring-ant. end	98-153	99-112	111-134	170-179	178-203	110-143	154-190	76^a^	95^a^
Excretory pore-ant. end	110-166	109-130	134-136	166-172	187-223	121-160	148-250	98^a^	112^a^
Pharynx length	156-195	159-178	167-176	220-225	189-284	177-218	206-265	250-279	218-247
Mid-body width	43-81	32-39	39-46	32-40	75-89	26-40	78-108	92-111	88-105
Anal body width	16-28	16-17	20-28	19-23	25-28	19-22	25-33	12-30	30-36
Tail length	68-101	65-80	75-96	77-87	115-129	81-100	94-112	100-148	84-132

**Notes:** ^a^Measurement obtained from illustrations. All measurements are in µm (except ratio and percentage) and in the form of range.

**Table 3.** Compendium of males of *Metarhabditis amsactae* (Ali, Pervez, Andrabi, Sharma and Verma, 2011) Sudhaus, 2011 populations and its synonyms.

Species	*M. amsactae*	*M. amsactae*	*M. amsactae* as *O. cicero*	*M. amsactae* as *O. hussainii*	*M. amsactae* as *O. gingeri*	*M. amsactae*	*M. amsactae* as *M. longicaudata*	*M. amsactae* as *M. rainai*	*Metarhabditis* sp. as *M. amsactae*
Reference	Present study	Ali et al. (2011)	Shaheen et al. (2011)	Shaheen et al. (2011)	Pervez et al. (2012)	Asif et al. (2013)	Tabassum et al. (2019)	Tabassum et al. (2019)	Tabassum et al. (2019)
Country	India	India	Pakistan	Pakistan	India	India	Pakistan	Pakistan	Pakistan
Habitat	Rhizosphere of sugarcane and groundnut	Rhizosphere of mungbean	Rhizosphere of chickpea	Rhizosphere of pigeonpea	Rhizosphere of ginger	Decaying matter	Rhizosphere of mango tree	Descomposed guava fruit	Rhizosphere of chicko
*L*	653-999	594-804	754-973	855-889	673-821	683-868	1,154-1,325	1,100-1,392	1,234-1,498
*a*	12.7-20.7	16.6-19.3	19.4-20.8	25.0.-28.0	18.3-24.0	18.1-21.7	14.4-19.4	15.0-24.0	14.0-20.0
*b*	3.8-5.9	4.0-5.0	5.0-5.6	3.83-3.89	4.32-5.3	4.3-4.5	5.3-6.6	4.0-6.0	6.0-8.0
*c*	9.8-20.9	10.7-17.8	13.6-16.7	13.9-13.6	11.5-16.7	11.5-13.7	14.9-19.0	23.0-37.0	16.0-20.0
*c′*	1.5-3.8	2.6-2.8	2.1-2.7	3.2	2.8-3.1	2.5-3.0	2.1-3.7	1.0-2.0	2.0-3.0
Lip region width	6-10	7-8	8-11	6-8	7^a^	9-10	11-14	?	?
Stoma length	13-21	15-17	19	22-23	17-19	18-20	20-28	24-28	20-24
Corpus length	58-76	81-109	?	134	71-112	72^a^	?	?	?
Isthmus length	35-41	27-42	?	44-56	21-38	27^a^	?	?	?
Bulb length	24-35	20-36	?	33-45	?	23^a^	?	?	?
Nerve ring-ant. end	92-125	79-108	116-136	149-179	90-114	100-127	143-185	?	?
Excretory pore-ant. end	113-144	87-114	127-138	155-168	110-142	119-141	137-179	?	?
Pharynx length	141-175	134-169	149-172	223-228	142-187	155-190	184-256	211-256	204-236
Midbody width	40-66	31-45	39-46	30-35	32-39	26-40	64-69	54-88	70-98
Anal body width	17-27	16-20	26-30	19-21	16-19	20-24	21-40	26-34	27-32
Tail length	49-62	41-55	55-58	61-65	43-59	58-67	62-76	32-56	66-78
Spicules length (SL)	34-49	31-36	35-44	41-44	24-27	33-39	40-46	32-60	42-60
Gubernaculum length (GL)	16-28	13-17	19-20	14-18	9-10	14-20	20-34	13-23	16-22
GL/SL × 100	50-60	43-46	45-54	34-40	36	42-51	50-74	40	37-38

**Notes:** ^a^Measurement obtained from illustrations; ? = Measurement unknown. All measurements are in µm (except ratio and percentage) and in the form of range.

**Table 4.** Nematode species, GenBank accession number, and origin of the sequences used for phylogenetic study.

	GenBank accession number					
Species^a^	18S rDNA	28S rDNA	ITS rDNA	Country	Isolation source	Reference
*Ablechroiulus cristatus*	EU196013	EU195976		USA	Unknown	Kiontke et al. (2007)
*Ablechroiulus dudichi*	AF083012			USA	Unknown	Fitch (unpublished)
*Auanema rhodensis*	EU196004			USA	Unknown	Kiontke et al. (2007)
*Buetschlinema nidrosiensis*	EU196020			USA	Unknown	Kiontke et al. (2007)
*Bursilla belari*	MK359049			India	Soil	Palanisamy et al. (unpublished)
*Bursilla* sp.		EF990722		USA	Unknown	Kiontke et al. (2007)
*Caenorhabditis angaria*	JN636068			USA	Rotting fruits	Kiontke et al. (2011)
*Caenorhabditis elegans*	Z92784			UK	Unknown	Sulston and Waterston (1998)
*Caenorhabditis elegans*		X03680		USA	Unknown	Ellis et al. (1986)
*Caenorhabditis sinica*		JN636142		USA	Rotting fruits	Kiontke et al. (2011)
*Cephaloboides cf. armatus*	EU196005			USA	Unknown	Kiontke et al. (2007)
*Crustorhabditis transita*		EU195995		USA	Unknown	Kiontke et al. (2007)
*Cruznema tripartitum*	U73449				Unknown	Baldwin et al. (1997)
*Diploscapter coronatus*	AY593921			Netherlands	Soil	Helder et al. (2006)
*Diploscapter* sp.		EU195959		USA	Unknown	Kiontke et al. (2007)
*Distolabrellus veechi*		EF990725		USA	Unknown	Kiontke et al. (2007)
*Haematozoon subulatum*	EU040125			China	Soil	Li et al. (unpublished)
*Haematozoon subulatum*	AF083017			USA	Unknown	Fitch (unpublished)
*Haematozoon subulatum*		EF990727		USA	Unknown	Kiontke et al. (2007)
*Heterorhabditis bacteriophora*	KJ636408			Netherlands	Unknown	van Megen (unpublished)
*Heterorhabditis downesi*			KU573061	Ireland	Soil	Maher et al. (2016)
*Heterorhabditis megidis*	KJ636320			Netherlands	Unknown	van Megen (unpublished)
*Litoditis marina*	AF083021			USA	Unknown	Fitch (unpublished)
*Litoditis marina*		AM399053		Belgium	*Fucus* sp.	Derycke et al. (2008a)
*Litoditis marina*			AM937053	Greece	Decaying seaweeds	Derycke et al. (2008b)
*Litoditis mediterranea*	AF083020			USA	Unknown	Fitch (unpublished)
*Litoditis mediterranea*		AM399068		New Zealand	Unknown	Derycke et al. (2008a)
*Mesorhabditis anisomorpha*	AF083013			USA	Unknown	Fitch (unpublished)
*Mesorhabditis anisomorpha*		EF990723		USA	Unknown	Kiontke et al. (2007)
*Mesorhabditis longespiculosa*	EU196014	EU195980		USA	Unknown	Kiontke et al. (2007)
*Metarhabditis amsactae*	MT872503, MT872504	MT872508, MT872509	MT873043, MT873044	India	Soil	Present study
*Metarhabditis amsactae*			MT422254	Philippines	Soil	Dichusa et al. (unpublished)
*Metarhabditis amsactae*			MT452472, MT452471	Philippines	Soil	Sangcopan and Sumaya (unpublished)
*Metarhabditis amsactae*			MH392568, KY083045 KP834433, KP834432	India	Soil	Chavan et al. (unpublished)
*Metarhabditis amsactae*			MK973071	Pakistan	Soil	Tabassum et al. (unpublished)
*Metarhabditis blumi*	U13935			Germany	Unknown	Fitch et al. (1995)
*Metarhabditis blumi*	MT043860			Philippines	Soil	Andalan et al. (unpublished)
*Metarhabditis blumi*	MF989442			Colombia	*Bos indicus*	Chaves et al. (unpublished)
*Metarhabditis blumi*	MT012150			Philippines	Soil compost	Guadalquiver (unpublished)
*Metarhabditis blumi*	MN453373			India	Soil	Kandhasamy and Muthugounder (unpublished)
*Metarhabditis blumi*		EU195965		USA	Unknown	Kiontke et al. (2007)
*Metarhabditis blumi*		KM233152, KM233153		Brazil	Cow	Bossi et al. (2015)
*Metarhabditis blumi*			DQ121436	South Africa	Unknown	Jumba and Gray (unpublished)
*Metarhabditis rainai*	MT012135, MT012133			Philippines	Soil	Kabalu et al. (unpublished)
*Metarhabditis rainai*	MT012153			Philippines	Soil	Buldiman et al. (unpublished)
*Metarhabditis rainai*	JQ237848			USA	Citrus groves	Campos-Herrera et al. (2012)
*Metarhabditis rainai*	AF083008			USA	Unknown	Fitch (unpublished)
*Metarhabditis rainai*		EU195966		USA	Unknown	Kiontke et al. (2007)
*Metarhabditis rainai*		KR011843, KR011846		Brazil	Soil	de Brida et al. (2017)
*Metarhabditis* sp.			MK973071	Pakistan	Soil	Tabassum et al. (unplublished)
*Myolaimus byersi*	KU180665	KU180676		Sweden	Unknown	Holovachov et al. (2015)
*Oscheius carolinensis*	FJ547240	FJ547239		USA	Vermicompost	Ye et al. (2010)
*Oscheius chongmingensis*	EF503692			China	Soil	Zhang et al. (2008)
*Oscheius chongmingensis*		EU273599		China	Soil	Liu et al. (2012)
*Oscheius chongmingensis*			MT548593	China	*Spodoptera frugiperda*	Li et al. (unpublished)
*Oscheius colombianus*	AY751546			Colombia	*Cyrtomenus berg*i	Stock et al. (2005)
*Oscheius dolichura*	EU196010			USA	Unknown	Kiontke et al. (2007)
*Oscheius dolichuroides*	AF082998			USA	Unknown	Fitch (unpublished)
*Oscheius dolichuroides*		EU195970		USA	Unknown	Kiontke et al. (2007)
*Oscheius guentheri*	EU196022			USA	Unknown	Kiontke et al. (2007)
*Oscheius indicus*		MF441252		India	Soil	Kumar et al. (2019)
*Oscheius insectivorus*	AF083019			USA	Unknown	Fitch (unpublished)
*Oscheius insectivorus*		EU195968		USA	Unknown	Kiontke et al. (2007)
*Oscheius myriophilus*		AY602176		USA	Unknown	Kiontke et al. (2004)
*Oscheius myriophilus*			MG742117	Thailand	Soil	Nitjarunkul et al. (unpublished)
*Oscheius myriophilus*	U13936			USA	Soil	Fitch et al. (1995)
*Oscheius necromenus*		KT884894		Australia	*Oncocladosoma castaneum*	Carta et al. (2018)
*Oscheius onirici*	MG551687			Portugal	Unknown	Campos-Herrera (unpublished)
*Oscheius onirici*		LN613263		Italy	Soil	Torrini et al. (2015)
*Oscheius rugaoensis*	JQ002566			China	Soil	Zhang et al. (2012)
*Oscheius rugaoensis*		KT884891		Japan	*Chamberlinius hualienensis*	Carta et al. (2018)
*Oscheius saproxylicus*	MK959600	MK959601		Spain	Decaying wood	Abolafia and Pena-Santiago (2019b)
*Oscheius tipulae*		EU195969		USA	Unknown	Kiontke et al. (2007)
*Oscheius tipulae*			KP792649	Brazil	Soil	Campos-Herrera and Pu˚ža (unpublished)
*Parasitorhabditis obtusa*		EF990724		Germany	Bark beetles	Kiontke et al. (2007)
*Parasitorhabditis obtuse*	EU003189			USA	Unknown	Kiontke et al. (2007)
*Pellioditis typica*			AF036946	Kenya	Feces of an antelope	Adams et al. (1998)
*Pelodera pseudoteres*	EU196023	EU195997		USA	Unknown	Kiontke et al. (2007)
*Pelodera teres*	AF083002			USA	Unknown	Fitch (unpublished)
*Pelodera teres*		EU195979		USA	Unknown	Kiontke et al. (2007)
*Phasmarhabditis hermaphrodita*	DQ639980			Scotland	Nemaslug	MacMillan et al. (2006)
*Phasmarhabditis neopapillosa*	DQ639982			Scotland	*Arion ater*	MacMillan et al. (2006)
*Phasmarhabditis papillosa*			KX267675	South Africa	*Deroceras reticulatum*	Pieterse et al. (2017)
*Poikilolaimus floridensis*	AB370214			USA	Termites	Kanzaki et al. (2009)
*Poikilolaimus oxycercus*	AF083023			USA	Unknown	Fitch (unpublished)
*Poikilolaimus oxycercus*		EU195984		USA	Unknown	Kiontke et al. (2007)
*Poikilolaimus piniperdae*		DQ059060		Germany	Unknown	Hong et al. (2005)
*Poikilolaimus regenfussi*	AF083022			USA	Unknown	Fitch (unpublished)
*Poikilolaimus regenfussi*		DQ059057		Germany	Unknown	Hong et al. (2005)
*Protorhabditis* sp.	AF083001			USA	Unknown	Fitch (unpublished)
*Protorhabditis* sp.		AY602168		USA	Unknown	Kiontke et al. (2007)
*Rhabditella axei*	U13934			Germany	Unknown	Fitch et al. (1995)
*Rhabditella axei*		AY602177		USA	Unknown	Kiontke et al. (2004)
*Rhabditis brassicae*	EU196006			USA	Unknown	Kiontke et al. (2007)
*Rhabditis* sp.	MN082353, MN082355			Taiwan	Soil	Yang et al. (2020)
*Rhabditoides inermis*	AF082996				Unknown	Fitch (unpublished)
*Rhabditoides regina*		EF990726		USA	Unknown	Kiontke et al. (2007)
*Rhomborhabditis regina*	KX385908			Mexico	White grub	Jiménez-Cortés et al. (2016)
*Teratorhabditis mariannae*	EF990716	EF990721		USA	Soil compost	Kanzaki et al. (2008)
*Teratorhabditis palmarum*	U13937			USA	Unknown	Fitch et al. (1995)

**Note:** ^a^Species names have been updated according their current nomenclature.

**Figure 4:**
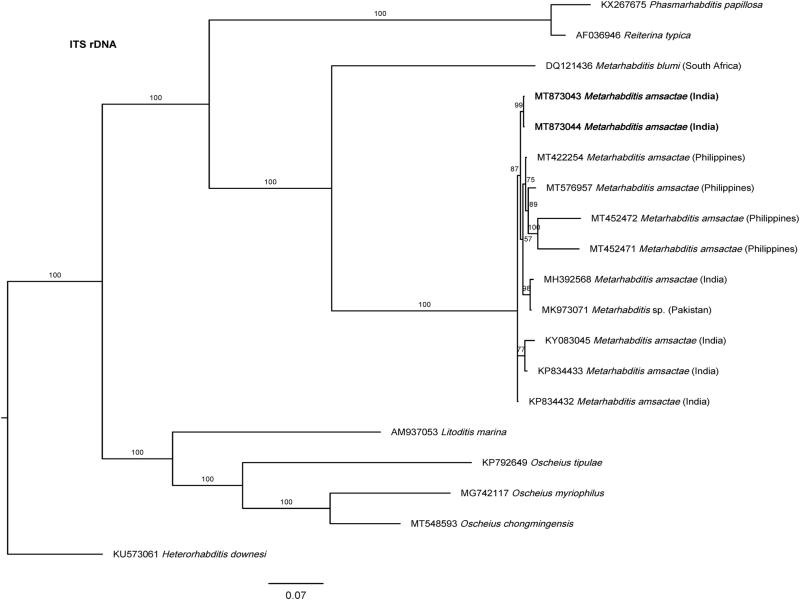
Bayesian Inference tree from previously and the newly sequenced *Metarhabditis amsactae* (bold) and other closely related species based on sequences of the Internal Transcribed Spacer (ITS1-5.8S-ITS2) rDNA region. Bayesian posterior probabilities (%) are given for each clade. The scale bar shows the number of substitutions per site.

Recently, Tabassum et al. (2019) described a new species, *Metarhabditis longicaudata* Tabassum, Salma and Nasir, 2019 from Pakistan. According to its morphology, especially males with posterior filiform part well developed, robust, and bursa posteriorly appearing parallel along it at its proximal part (unfortunately, the LM Fig. 2C, D provided by these authors seems to be strongly stretched making the stoma too much long and narrow, and not agreeing with the line drawing Fig. 1C, D provided by these same authors), and morphometric characteristics (Tables 1 and 2), the specimens described are highly similar to *M. amsactae*. Given these considerations, we propose that *Metarhabditis longicaudata* is a junior synonym of *Metarhabditis amsactae*. Moreover, the specimens described as *M. amsactae* in the same study by these Pakistani authors do not present characteristics of this species, especially because the males lack posterior filiform part of the tail, spicules lack ventral bent or hooked manubrium, and females have a long rectum. In addition, the nematode population described as *M. rainai* (Carta and Osbrink, 2005) Sudhaus, 2011 in the same study by Tabassum et al. (2019) are morphologically very similar to *M. amsactae* nematodes (Tables 1 and 2) and, hence they were misidentified. ITS-phylogenetic trees support these conclusions as the sequences submitted to GenBank by these authors, *Metarhabditis* sp. (MK973071), cluster together with other *M. amsactae* (Fig. 4).

According to this, the updated list of synonyms of *Metarhabditis amsactae* is as follows:

*Metarhabditis amsactae* (Ali, Pervez, Andrabi, Sharma and Verma, 2011) Sudhaus, 2011

= *Oscheius amsactae* Ali, Pervez, Andrabi, Sharma and Verma, 2011

= *Oscheius ciceri* Shaheen, Ali and Asif, 2011

= *Oscheius hussainii* Shaheen, Ali and Asif, 2011

= *Oscheius gingeri* Pervez, Eapen, Devasahayan and Jacob, 2012

= *Metarhabditis longicaudata* Tabassum, Salma and Nasir, 2019

### Phylogenetic relationships

**Figure 5:**
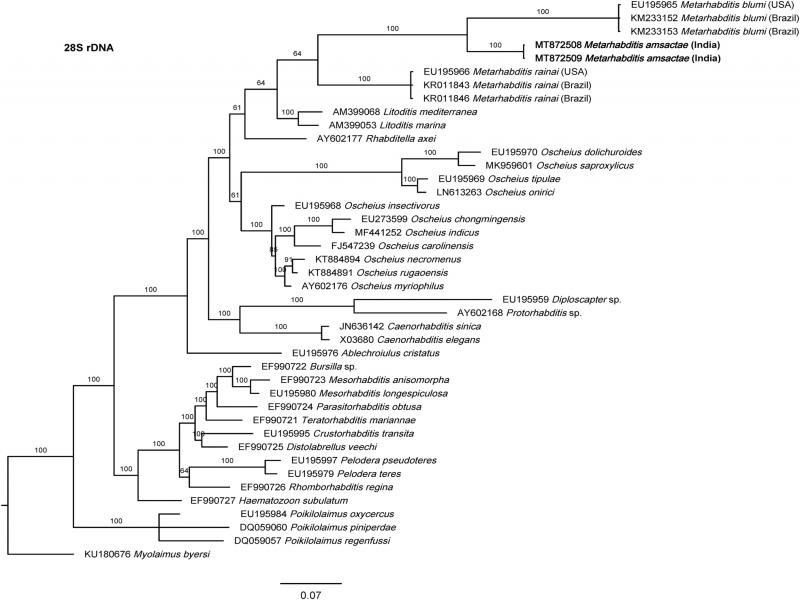
Bayesian Inference tree from the newly sequenced *Metarhabditis amsactae* (bold) and other closely related species based on sequences of the small subunit (18S) of rDNA region. Bayesian posterior probabilities (%) are given for each clade. The scale bar shows the number of substitutions per site.

**Figure 6:**
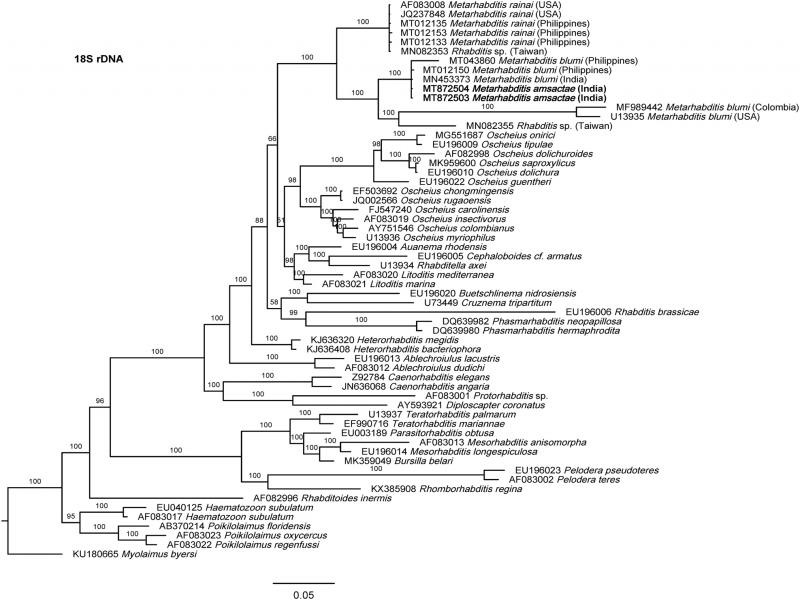
Bayesian Inference tree from the newly sequenced *Metarhabditis amsactae* (bold) and other closely related species based on sequences of the D2/D3 domain of large subunit (28S) of rDNA region. Bayesian posterior probabilities (%) are given for each clade. The scale bar shows the number of substitutions per site.

The phylogenetic relationships as inferred from the Bayesian Inference analysis between *Metarhabditis amsactae* and other closely related are provided based on ITS- (Fig. 4), 18S- (Fig. 5), and 28S- (Fig. 6) rDNA fragments. Based on the three phylogenetic trees, *M. blumi* (Sudhaus, 1974) Sudhaus, 2011 and *M. rainai* (Carta and Osbrink, 2005) Sudhaus, 2011 are sister species of *M. amsactae.*

The phylogenetic tree inferred using 18S rDNA gene sequences, shows three clusters that contain sequences of nematodes that have been suggested to belong to *Metarhabditis* (Fig. 5). One cluster is composed of *M. rainai* (AF083008, JQ237848, MT012133, MT012135 and MT012153) and one nematode isolate that might have been misidentified as *Rhabditis* sp (MN082353) but could correspond to *M. rainai*. A second cluster composed of *M. amsactae* (MT872504, MT872503) and three nematode isolates that might have been misidentified as *M. blumi* (MT043860, MT012150, MN453373). A third cluster composed of *M. blumi* (MF989442, U13935), and *Rhabditis* sp. (MN082355). As *M. amsactae* isolates that correspond to accession numbers MT872504 and MT872503, and the *M. blumi* isolate that correspond to accession numbers U13935 have been morphologically and molecularly characterized, we conclude that nematodes isolates with NCBI accessions MT043860, MT012150 and MN453373 are actually *M. amsactae* instead of *M. blumi*, and the nematodes isolate with NCBI accession MN082355 identified as *Rhabditis* sp. should correspond to *Metarhabditis* sp. This conclusion is also supported by sequence identity analysis. Comparing the nucleotide composition of a common 18S rDNA gene fragment of 723 bp in length of the *M. amsactae* specimens examined in this study (MT872503-4) and the nucleotide composition of *M. blumi* (U13935), *Rhabditis* sp. (MN082355), *M. rainai* (AF083008, JQ237848, MT012133, MT012135 and MT012153), *Rhabditis* sp. (MN082353), and *M. blumi* (MT043860, MT012150 and MN453373), we found 57 genetic changes (insertions, deletions or substitutions) between *M. amsactae* and *M. blumi*, 69 genetic changes between *M. amsactae* and *Rhabditis* sp. (MN082355), 82 changes between *M. amsactae* and *M. rainai*, 82 changes between *M. amsactae* and *Rhabditis* sp. (MN082353), 95 changes between *M. blumi* and *M. rainai*, and fewer genetic changes between *Rhabditis* sp. (MN082353) and *M. rainai*, and between the *M. amsactae* (MT872503-4) and *M. blumi* (MT043860, MT012150 and MN453373).

The phylogenetic tree inferred using 28S rDNA gene sequences show also three clusters containing *Metarhabditis* nematodes: one with *M. blumi*, one with *M. amsactae*, and one *M. rainai* (Fig. 6). Comparing the nucleotide composition of a common 28S rDNA fragment of 343 bp in length of the *M. amsactae* specimens examined in this study (MT872508-9) and the nucleotide composition of *M. blumi* (EU195965, KM233152-3), and *M. rainai* (EU195966, KR011843-6), we found 75 genetic changes (insertions, deletions or substitutions) between *M. amsactae* and *M. blumi*, 80 genetic changes between *M. amsactae* and *M. rainai*, and 95 changes between *M. blumi* and *M. rainai*, suggesting that *M. amsactae, M. rainai* and *M. blumi* are sister species and that the nematode isolates characterized in this study belong to *M. amsactae*.

Finally, analyzing ITS rRNA gene sequences, we arrive to the same conclusions derived from the analysis of 28S- and 18S rDNA gene sequences. ITS-based phylogenetic tree show a clear cluster that separates *M. amsactae* and *M. blumi* (Fig. 4). Unfortunately, there are no available *M. rainai* sequences. Sequence comparisons show that a common ITS rDNA fragment of 802 bp in length of *M. amsactae* (MT873043-4) and of *M. blumi* (DQ121436) differ in 496 changes, which again support the status of the nematode isolates of this study as *M. amsactae* (Table 4).

## References

[ref001] Aasha, R. , Chaubey, A. K. and Bhat, A. H. 2019. Notes on *Steinernema abbasi* (Rhabditida: Steinernematidae) strains and virulence tests against lepidopteran and coleopterans pests. Journal of Entomology and Zoology Studies 7:954–964.

[ref002] Abolafia, J. 2015. A low-cost technique to manufacture a container to process meiofauna for scanning electron microscopy. Microscopy Research and Technique 78:771–776. 26178782 10.1002/jemt.22538

[ref003] Abolafia, J. and Peña-Santiago, R. 2017. On the identity of *Chiloplacus magnus* (Rashid & Heyns, 1990) and *C. insularis* (Orselli & Vinciguerra, 2002) (Rhabditida: Cephalobidae), two confusable species. Nematology 19:1017–1034.

[ref004] Abolafia, J. and Peña-Santiago, R. 2019a. Description of *Metarhabditis giennensis* sp. n. (Nematoda, Rhabditida, Rhabditidae) from decaying wood of riverbank forest in the southern Iberian Peninsula. Zootaxa 4652:145–154. 10.11646/zootaxa.4652.1.8 31716888

[ref005] Abolafia, J. and Pena-Santiago, R. 2019b. Morphological and molecular characterization of *Oscheius saproxylicus* sp. n. (Rhabditida, Rhabditidae) from decaying wood in Spain, with new insights into the phylogeny of the genus and a revision of its taxonomy. Journal of Nematology 51:1–21. 10.21307/jofnem-2019-053 PMC6909031 34179804

[ref006] Adams, B. J. , Burnell, A. M. and Powers, T. O. 1998. A phylogenetic analysis of *Heterorhabditis* (Nematoda: Rhabditidae) based on internal transcribed spacer 1 DNA sequence data. Journal of Nematology 30:22–39. 19274196 PMC2620274

[ref007] Ali, S. S. , Pervez, R. , Andrabi, R. , Sharma, R. and Verma, V. 2011. *Oscheius amsactae* n. sp. (Nematoda: Rhabditida), a necromenic associate of red hairy caterpillar, *Amsacta moori* (Lepidoptera: Arctiidae) from Kanpur, India. Archives Phytopathology and Plant Protection 449:871–881.

[ref008] Altschul, S. F. , Gish, W. , Miller, W. , Myers, E. W. and Lipman, D. J. 1990. Basic local alignment search tool. Journal of Molecular Biology 215:403–410. 2231712 10.1016/S0022-2836(05)80360-2

[ref009] Andrássy, I. 1976. Evaluation as a basis for the systematization of nematodes Eotvos Lorand University, Budapest, Hungary, 288pp.

[ref010] Asif, M. , Prasad, J. S. , Khan, R. , Somasekhar, N. and Tahseen, Q. 2013. A revision of the genus *Metarhabditis* (Nematoda: Rhabditidae) with description of three known species, a key to the identification of congeners and their relationships. Journal of Natural History 47:41–42..

[ref011] Baldwin, J. G. , Frisse, L. M. , Vida, J. T. , Eddleman, C. D. and Thomas, W. K. 1997. An evolutionary framework for the study of developmental evolution in a set of nematodes related to *Caenorhabditis elegans* . Molecular Phylogenetics and Evolution 8:249–259. 9299229 10.1006/mpev.1997.0433

[ref012] Bedding, R. A. and Akhurst, R. J. 1975. A simple technique for the detection of insect parasitic nematodes in soil. Nematologica 21:109–110.

[ref013] Bharti, L. , Bhat, A. H. , Chaubey, A. K. and Abolafia, J. 2020. Morphological and molecular characterization of *Merlinius brevidens* (Allen, 1955) Siddiqi, 1970 (Nematoda, Rhabditida, Merlinidae) from India. Journal of Natural History 54:1477–1498.

[ref014] Bhat, A. H. , Chaubey, A. K. and Půža, V. 2018. The first report of *Xenorhabdus indica* from *Steinernema pakistanense*: co-phylogenetic study suggests co-speciation between *X. indica* and its steinernematid nematodes. Journal of Helminthology 92:1–10. 29338795 10.1017/S0022149X17001171

[ref015] Bhat, A. H. , Sharma, L. and Chaubey, A. K. 2020a. Characterisation of *Xenorhabdus stockiae* associated symbiont of *Steinernema surkhetense* with a note on its geographical distribution and virulence. Egyptian Academic Journal of Biological Sciences A. Entomology 13:105–122.

[ref016] Bhat, A. H. , Chaubey, A. K. , Shokoohi, E. and Machado, R. A. R. 2020c. Molecular and phenotypic characterization of *Heterorhabditis indica* (Nematoda: Rhabditida) nematodes isolated during a survey of agricultural soils in Western Uttar Pradesh, India 65:1–17.

[ref017] Bhat, A. H. , Chaubey, A. K. , Shokoohi, E. and Mashela, P. W. 2019. Study of *Steinernema hermaphroditum* (Nematoda, Rhabditida) from the West Uttar Pradesh, India. Acta Parasitologica 64:720–737. 31077031 10.2478/s11686-019-00061-9

[ref018] Bhat, A. H. , Askary, T. H. , Ahmad, M. J. , Suman, A. and Chaubey, A. K. 2020b. Description of *Heterorhabditis bacteriophora* (Nematoda: Heterorhabditida) isolated from hilly areas of Kashmir Valley. Egyptian Journal of Biological Pest Control 96:1–7.

[ref019] Bhat, A. H. , Istkhar, R. , Chaubey, A. K. , Půža, V. and San-Blas, E. 2017. First report and comparative study of *Steinernema surkhetense* (Rhabditida: Steinernematidae) and its symbiont bacteria from subcontinental India. Journal of Nematology 49:92–102. 28512381 10.21307/jofnem-2017-049 PMC5411258

[ref020] Bossi, P. V. , Consoli, E. A. , Rosa, J. M. O. , Leite, L. B. , Leite, R. C. and de Oliveira, C. M. G. 2015. Molecular identification and phylogenetic analysis of *Metarhabditis blumi* (Nematoda: Rhabditida). Veterinary Parasitology 214:184–186. 26464070 10.1016/j.vetpar.2015.06.014

[ref021] Campos-Herrera, R. , El-Borai, F. E. and Duncan, L. W. 2012. Wild interguild relationship among entomopathogenic and free–living nematodes in soil as a measured by real time qPCR. Journal of Invertebrate Pathology 111:126–135. 22841945 10.1016/j.jip.2012.07.006

[ref022] Carta, L. K. and Osbrink, W. 2005. *Rhabditis rainai* n. sp. (nematode: Rhabditida) associated with the formosan subterranean termite, *Coptotermes formosanus* (Isoptera: Rhinotermitidae). Nematology 7:863–879.

[ref023] Carta, L. K. , Thomas, W. K. and Meyer-Rochow, V. B. 2018. Two nematodes (Nematoda: Diplogastridae, Rhabditidae) from the invasive millipede *Chamberlinius hualienensis* Wang, 1956 (Diplopoda, Paradoxosomatidae) on Hachijojima Island in Japan. Journal of Nematology 50:479–486. 31094150 10.21307/jofnem-2018-048 PMC6909329

[ref024] Courtney, W. D. , Polley, D. and Miller, V. I. 1955. TAF, an improved fixative in nematode technique. Plant Disease Reporter 39:570–571.

[ref025] Darriba, D. , Taboada, G. L. , Doallo, R. and Posada, D. 2012. jModelTest 2: more models, new heuristics and parallel computing. Nature Methods 9:772. 10.1038/nmeth.2109 PMC4594756 22847109

[ref026] de Brida, A. L. , Rosa, J. M. , Oliveira, C. M. , Castro, B. M. , Serrão, J. E. , Zanuncio, J. C. , Leite, L. G. and Wilcken, S. R. 2017. Entomopathogenic nematodes in agricultural areas in Brazil. Scientific Reports 7:452–454. 28382937 10.1038/srep45254 PMC5382772

[ref027] De Ley, P. , van de Velde, M. C. , Mounport, D. , Baujard, P. and Coomans, A. 1995. Ultrastructure of the stoma in Cephalobidae, Panagrolaimidae and Rhabditidae, with a proposal for a revised stoma terminology in Rhabditida (Nematoda). Nematologica 41:153–182.

[ref028] de Man, J. G. 1881. Die einheimischen, frei in der reinen Erde und im süssen Wasser lebenden Nematoden monographisch bearbeitet. Tijdschrift der Nederlandsche Dierkundige Vereeniging 5:1–104.

[ref029] Derycke, S. , Fonseca, G. , Vierstraete, A. , Vanfleteren, J. , Vincx, M. and Moens, T. 2008a. Disentangling taxonomy within the *Rhabditis* (*Pellioditis*) *marina* (Nematoda, Rhabditidae) species complex using molecular and morhological tools. Zoological Journal of the Linnean Society 152:1–15.

[ref030] Derycke, S. , Remerie, T. , Backeljau, T. , Vierstraete, A. , Vanfleteren, J. , Vincx, M. and Moens, T. 2008b. Phylogeography of the *Rhabditis* (*Pellioditis*) *marina* species complex: evidence for long-distance dispersal, and for range expansions and restricted gene flow in the northeast Atlantic. Molecular Ecology 17:3306–3322. 18573165 10.1111/j.1365-294X.2008.03846.x

[ref031] Dujardin, F. 1845. Histoire naturelle des helminthes ou vers intestinaux. Librairie Encyclopédique de Roret, Paris: 654 pp. + 12 plates.

[ref032] Ellis, R. E. , Sulston, J. E. and Coulson, A. R. 1986. The rDNA of *C. elegans*: sequence and structure. Nucleic Acids Research 14:2345–2364. 3960722 10.1093/nar/14.5.2345 PMC339662

[ref033] Fitch, D. H. , Bugaj-Gaweda, B. and Emmons, S. W. 1995. 18S ribosomal RNA gene phylogeny for some Rhabditidae related to *Caenorhabditis* . Journal of Molecular Biology and Evolution 12:346–358. 7700158 10.1093/oxfordjournals.molbev.a040207

[ref034] Floyd, R. M. , Rogers, A. D. , Lambshead, P. J. D. and Smith, C. R. 2005. Nematode specific PCR primers for the 18S small subunit rRNA gene. Molecular Ecology Notes 5:611–612.

[ref035] Hall, T. A. 1999. Bioedit: a user-friendly biological sequence alignment editor and analysis program for Windows 95/98/NT. Nucleic Acids Symposium Series 41:95–98.

[ref036] Holovachov, O. , Camp, L. and Nadler, S. A. 2015. Sensitivity of ribosomal RNA character sampling in the phylogeny of Rhabditida. Journal of Nematology 47: 337–355. 26941463 PMC4755709

[ref037] Hong, R. L. , Villwock, A. and Sommer, R. J. 2005. Cultivation of the rhabditid *Poikilolaimus oxycercus* as a laboratory nematode for genetic analyses. Journal of Experimental Biology 303:742–760. 10.1002/jez.a.200 16106407

[ref038] Jiménez-Cortés, J. G. , Canales-Lazcano, J. , Lara-Reyes, N. , Rosenblueth, M. , Martinez-Romero, E. and Contreras-Garduno, J. 2016. Microbiota from *Rhabditis regina* may alter nematode entomopathogenicity. Parasitology Research 115:4153–4165. 27492201 10.1007/s00436-016-5190-3

[ref039] Kajol, Y. , Bhat, A. H. , Aasha, R. and Chaubey, A. K. 2020. Biochemical and molecular characterization of associated *Photorhabdus* symbiont of Indian strain of *Heterorhabditis indica* and its efficacy. Pakistan Journal of Nematology 38:15–24.

[ref040] Kanzaki, N. , Giblin-Davis, R. M. , Scheffrahn, R. H. and Center, B. J. 2009. *Poikilolaimus floridensis* n. sp. (Rhabditida: Rhabditidae) associated with termites (Kalotermitidae). Nematology 11:203–216.

[ref041] Kanzaki, N. , Kiontke, K. , Giblin-Davis, R. , Abe, F. , Soné, K. , Hata, K. and Fitch, D. 2008. *Teratorhabditis synpapillata* (Sudhaus 1985) (Rhabditida: Rhabditidae) is an associate of the red palm weevil, *Rhynchophorus ferrugineus* (Coleoptera: Curculionidae). Nematology 10:207–218.

[ref042] Kiontke, K. , Gavin, N. P. , Raynes, Y. , Roehrig, C. , Piano, F. and Fitch, D. H. 2004. *Caenorhabditis* phylogeny predicts convergence of hermaphroditism and extensive intron loss. Proceedings of National Academy of Sciences of the United States of America 101:9003–9008. 10.1073/pnas.0403094101 PMC428462 15184656

[ref076] Kiontke, K. , Barrière, A. , Kolotuev, I. , Podbilewicz, B. , Sommer, R. J. , Fitch, D. H. A. and Félix, M. A. 2007. Trends, stasis and drift in the evolution of nematode vulva development. Current Biolgy 17:1925–1937. 10.1016/j.cub.2007.10.061 18024125

[ref043] Kiontke, K. C. , Felix, M. A. , Ailion, M. , Rockman, M. V. , Braendle, C. , Penigault, J. B. and Fitch, D. H. 2011. A phylogeny and molecular barcodes for *Caenorhabditis*, with numerous new species from rotting fruits. BMC Evolutionary Biology 11:1–18. 22103856 10.1186/1471-2148-11-339 PMC3277298

[ref045] Kumar, P. , Jamal, W. , Somvanshi, V. S. , Chauban, K. and Mumtaz, S. 2019. Description of *Oscheius indicus* n. sp. (Rhabditidae: Nematoda) from India. Journal of Nematology 51:1–11. 10.21307/jofnem-2019-004 PMC6929651 31115203

[ref044] Kumar, S. , Stecher, G. and Tamura, K. 2016. MEGA7: Molecular Evolutionary Genetics Analysis Version 7.0 for bigger datasets. Molecular Biology and Evolution 33:1870–1874. 27004904 10.1093/molbev/msw054 PMC8210823

[ref046] Liu, Q. Z. , Mráček, Z. , Zhang, L. J. , Půža, V. and Dong, L. M. 2012. Re-description of *Oscheius chongmingensis* (Zhang et al., 2008) (Nematoda: Rhabditidae) and its entomopathogenicity. Nematology 14:139–149.

[ref047] MacMillan, K. , Blok, V. , Young, I. , Crawford, J. and Wilson, M. J. 2006. Quantification of the slug parasitic nematode *Phasmarhabditis hermaphrodita* from soil samples using real time qPCR. International Journal of Parasitology 36:1453–1461. 17010977 10.1016/j.ijpara.2006.08.005

[ref048] Maher, A. M. D. , Asaiyah, M. A. M. , Brophy, C. and Griffin, C. T. 2016. An entomopathogenic nematode extends its niche by associating with different symbionts. Journal of Microbial Ecology 73:211–223. 27543560 10.1007/s00248-016-0829-2

[ref049] Martins, W. 1985. *Rhabditis* (*Rhabditis*) *freitasi* sp. n. e *Rhabditis* (*Rhabditis*) *costai* sp. (Nematoda: Rhabditidae) isolados de otite bovina. Memórias do Instituto Oswaldo Cruz 80:11–16. 4088041

[ref050] Nadler, S. A. , Bolotin, E. and Stock, S. P. 2006. Phylogenetic relationships of *Steinernema* Travassos, 1927 (Nematoda: Cephalobina: Steinernematidae) based on nuclear, mitochondrial and morphological data. Systematic Parasitology l63:161–181. 10.1007/s11230-005-9009-3 16541298

[ref051] Örley, L. 1880. Az Anguillulidák magánrajza. (Monographie der Anguilluliden). Természetraji Füzetek 4:16–150.

[ref052] Pervez, R. , Eapen, S. J. , Devasahayan, S. and Jacob, T. K. 2012. A new species of entomopathogenic nematode *Oscheius gingeri* sp. n. (Nematoda: Rhabditidae) from ginger rhizosphere. Archives of Phytopathology and Plant Protection 5:526–535.

[ref053] Pieterse, A. , Tiedt, L. R. , Malan, A. P. and Ross, J. L. 2017. First record of *Phasmarhabditis papillosa* (Nematoda: Rhabditidae) in South Africa, and its virulence against the invasive slug, *Deroceras panormitanum* . Nematology 19:1035–1050.

[ref054] Poinar, G. O. Jr. 1971. *Rhabditis adenobia* sp. n. (Nematoda: Rhabditidae) from the colleterial glands of *Oryctes monoceros* L. and other tropical dynastid beetles (Coleoptera: Scarabaeidae). Proceedings of the Helminthological Society of Washington 38:99–108.

[ref055] Rambaut, A. 2018. FigTree, a graphical viewer of phylogenetic trees (Version 1.4.4). available at http://tree.bio.ed.ac.uk/software/figtree.

[ref056] Rana, A. , Bhat, A. H. , Chaubey, A. K. , Bhargava, S. and Abolafia, J. 2020a. Morphological and molecular characterization of *Acrobeloides saeedi* Siddiqi, De Ley and Khan, 1992 (Rhabditida, Cephalobidae) from India and comments on its status. Journal of Nematology 52:e2020–27. 10.21307/jofnem-2020-027 PMC7266034 32342680

[ref057] Rana, A. , Bhat, A. H. , Chaubey, A. K. , Shokoohi, E. and Richardo, M. 2020b. Morphological and molecular characterization *Heterorhabditis bacteriophora* nematodes isolated from Indian agricultural soils and their biocontrol potential. Zootaxa 4878:77–102. 10.11646/zootaxa.4878.1.3 33311167

[ref058] Ronquist, F. , Teslenko, M. , van der Mark, P. , Ayres, D. L. , Darling, A. , Höhna, S. , Larget, B. , Liu, L. , Suchard, M. A. and Huelsenbeck, J. P. 2012. MrBayes 3.2: efficient Bayesian phylogenetic inference and model choice across a large model space. Systematic Biology 61:539–542. 22357727 10.1093/sysbio/sys029 PMC3329765

[ref059] Seinhorst, J. W. 1959. A rapid method for the transfer of nematodes from fixative to anhydrous glycerin. Nematologica 4:67–69.

[ref060] Shaheen, A. , Ali, S. S. and Asif, M. 2011. Two new species of genus *Oscheius* from pulses ecosystem in Uttar Pradesh, India. Trends in Biosciences 4:82–85.

[ref061] Stock, S. P. , Caicedo, A. M. and Calatayud, P. A. 2005. *Rhabditis* (*Oscheius*) *colombiana* n. sp. (Nematoda: Rhabditida), a necromenic associate of the subterranean burrower bug *Cyrtomenus bergi* (Hemiptera: Cydnidae) from the Cauca Valley, Colombia. Nematology 7:363–373.

[ref062] Sudhaus, W. 1974. Zur Systematik, Verbreitung, Ökologie und Biologie neuer und wenig bekannter Rhabditiden (Nematoda). Teil, Zoologische Jahrbücher Systematik 101:173–212.

[ref063] Sudhaus, W. 2011. Phylogenetic systematisation and catalogue of paraphyletic “Rhabditidae” (Secernentea, Nematoda). Journal of Nematode Morphology and Systematics 14:113–178.

[ref064] Sulston, J. and Waterston, R. 1998. Genomic sequence of the nematode *C. elegance:* a plateform for investigating biology. Science 283:2012–2018. 10.1126/science.282.5396.2012 9851916

[ref065] Suman, B. , Bhat, A. H. , Aasha, R. , Chaubey, A. K. and Abolafia, J. 2020. Morphological and molecular characterisation of *Distolabrellus veechi* (Rhabditida: Mesorhabditidae) from India. Nematology 22:439–452.

[ref067] Tabassum, A. K. , Salma, J. and Nasir, M. 2019. Description of new species of *Metarhabditis longicaudata* (Nematoda: Rhabditidae) with three new records from Sindh, Pakistan. Plant Protection 3:131–139.

[ref068] Tahseen, Q. , Hussain, A. , Tomar, V. , Shah, A. A. and Jairajpuri, M. S. 2004. Description of *Metarhabditis andrassyana* gen. sp. n. (Nematoda: Rhabditidae) from India. International Journal of Nematology 14:163–168.

[ref069] Torrini, G. , Mazza, G. , Carletti, B. , Benvenuti, C. , Roversi, P. F. , Fanelli, E. , De Luca, F. , Troccoli, A. and Tarasco, E. 2015. *Oscheius onirici* sp. n. (Nematoda: Rhabditidae): a new entomopathogenic nematode from an Italian cave. Zootaxa 3937:533–548. 25947484 10.11646/zootaxa.3937.3.6

[ref070] Vrain, T. C. , Wakarchuk, D. A. , Levesque, A. C. and Hamilton, R. I. 1992. Intra-specific rDNA restriction fragment length polymorphisms in the *Xiphinema americanum* group. Fundamental and Applied Nematology 15:563–574.

[ref071] White, G. F. 1927. A method for obtaining infective nematode larvae from cultures. Science 66:302–303. 10.1126/science.66.1709.302-a 17749713

[ref072] Yang, C. T. , de Ulzurrun, G. V. D. , Gonçalves, P. , Lin, H. C. , Chang, C. W. , Huang, T. Y. , Chen, S. A. , Lai, C. K. , Tsai, I. J. , Schroeder, F. C. , Stajich, J. E. and Hsueh, Y. P. 2020. Natural diversity in the predatory behavior facilitates the establishment of a robust model strain for nematode-trapping fungi. Proceedings of the National Academy of Sciences 117:6762–6770. 10.1073/pnas.1919726117 PMC7104180 32161129

[ref073] Ye, W. , Torres-Barragan, A. and Cardoza, Y. J. 2010. *Oscheius carolinensis* n. sp. (Nematoda: Rhabditidae), a potential entomopathogenic nematode from vermicompost. Nematology 12:121–135.

[ref074] Zhang, C. , Liu, J. , Xu, M. , Sun, J. , Yang, S. , An, X. , Gao, G. , Lin, M , Lai, R. , He, Z. , Wu, Y. and Zhang, K. 2008. *Heterorhabditidoides chongmingensis* gen. nov., sp. nov. (Rhabditida: Rhabditidae), a novel member of the entomopathogenic nematodes. Journal of Invertebrate Pathology 98:153–168. 18410943 10.1016/j.jip.2008.02.011

[ref075] Zhang, K. Y. , Liu, X. H. , Tan, J. , Wang, Y. , Qiao, L. , Yedid, G. , Dai, C. S. , Qiu, R. L. , Yan, X. W. , Tan, H. W. , Su, Z. Y. , Lai, R. and Gao, G. F. 2012. *Heterorhabditidoides rugaoensis* n. sp. (Rhabditida: Rhabditidae), a novel highly pathogenic entomopathogenic nematode member of Rhabditidae. Journal of nematology 44:348–360. 23482845 PMC3592371

